# Establishment and validation of early prediction model for hypertriglyceridemic severe acute pancreatitis

**DOI:** 10.1186/s12944-023-01984-z

**Published:** 2023-12-08

**Authors:** Yi Shuanglian, Zeng Huiling, Lin Xunting, Deng Yifang, Lin Yufen, Xie Shanshan, Si Lijuan, Liu Yunpeng

**Affiliations:** 1 Department of Gastroenterology, The National Key Clinical Specialty, Zhongshan Hospital of Xiamen University, School of Medicine, Xiamen University, Xiamen, Fujian Province 361004 P. R. China; 2 Xiamen Key Laboratory of Intestinal Microbiome and Human Health, Zhongshan Hospital of Xiamen University, School of Medicine, Xiamen University, Xiamen, Fujian Province 361004 P. R. China; 3 Institute for Microbial Ecology, School of Medicine, Xiamen University, Xiamen, Fujian Province 361004 P. R. China; 4 Department of Digestive Disease, School of Medicine, Xiamen University, Xiamen, Fujian Province 361004 P. R. China

**Keywords:** Hypertriglyceridemia, Acute pancreatitis, Prediction model, Severity

## Abstract

**Background:**

The prevalence of hypertriglyceridaemia-induced acute pancreatitis (HTG-AP) is increasing due to improvements in living standards and dietary changes. However, currently, there is no clinical multifactor scoring system specific to HTG-AP. This study aimed to screen the predictors of HTG-SAP and combine several indicators to establish and validate a visual model for the early prediction of HTG-SAP.

**Methods:**

The clinical data of 266 patients with HTG-SAP were analysed. Patients were classified into severe (N = 42) and non-severe (N = 224) groups according to the Atlanta classification criteria. Several statistical analyses, including one-way analysis, least absolute shrinkage with selection operator (LASSO) regression model, and binary logistic regression analysis, were used to evaluate the data.

**Results:**

The univariate analysis showed that several factors showed no statistically significant differences, including the number of episodes of pancreatitis, abdominal pain score, and several blood diagnostic markers, such as lactate dehydrogenase (LDH), serum calcium (Ca^2+^), C-reactive protein (CRP), and the incidence of pleural effusion, between the two groups (*P* < 0.000). LASSO regression analysis identified six candidate predictors: CRP, LDH, Ca^2+^, procalcitonin (PCT), ascites, and Balthazar computed tomography grade. Binary logistic regression multivariate analysis showed that CRP, LDH, Ca^2+^, and ascites were independent predictors of HTG-SAP, and the area under the curve (AUC) values were 0.886, 0.893, 0.872, and 0.850, respectively. The AUC of the newly established HTG-SAP model was 0.960 (95% confidence interval: 0.936–0.983), which was higher than that of the bedside index for severity in acute pancreatitis (BISAP) score, modified CT severity index, Ranson score, and Japanese severity score (JSS) CT grade (AUC: 0.794, 0.796, 0.894 and 0.764, respectively). The differences were significant (*P* < 0.01), except for the JSS prognostic indicators (*P* = 0.130). The Hosmer–Lemeshow test showed that the predictive results of the model were highly consistent with the actual situation (*P* > 0.05). The decision curve analysis plot suggested that clinical intervention can benefit patients when the model predicts that they are at risk for developing HTG-SAP.

**Conclusions:**

CRP, LDH, Ca^2+^, and ascites are independent predictors of HTG-SAP. The prediction model constructed based on these indicators has a high accuracy, sensitivity, consistency, and practicability in predicting HTG-SAP.

**Supplementary Information:**

The online version contains supplementary material available at 10.1186/s12944-023-01984-z.

## Background

Acute pancreatitis (AP) is caused by the premature activation of pancreatic enzymes, leading to the digestion of the pancreatic tissue and inflammation or necrosis of the pancreas and surrounding tissues. It can even progress to a systemic failure of one or more organs and is an unpredictable and potentially fatal common digestive disease [1, 2]. According to Chinese guidelines, gallstone disease remains the predominant aetiology of AP. However, it is crucial to recognise that the prevalence of hypertriglyceridaemia-induced acute pancreatitis (HTG-AP) is increasing, attributable to improvements in living standards and dietary changes, and has even surpassed alcohol as the second most frequent cause of AP [3–6]. HTG-AP has worse clinical outcomes than AP associated with other aetiologies. A recent systematic review and meta-analysis revealed significantly higher odds ratios for persistent systemic inflammatory response syndrome (SIRS), continuous organ failure, and mortality among patients with HTG-AP [7]. The severity of AP is categorised as mild, moderate, or severe according to the revised Atlanta classification [8]. Severe AP (SAP), with reported mortality rates of 30% or higher, causes significantly greater morbidity and mortality than moderate SAP; meanwhile, persistent organ failure lasting more than 48 h is associated with a mortality rate of approximately 50% [9–11]. Therefore, early recognition of patients at a higher risk of developing complications is necessary to reduce the risk of adverse disease outcomes and death.

The clinical manifestations of HTG-AP are similar to those of AP induced by other aetiologies, usually presenting as acute, persistent mid-upper abdominal pain radiating to the lower back, accompanied by symptoms such as nausea, vomiting, and mild fever in some patients [5]. However, in HTG-AP, alongside pancreatic function and imaging abnormalities, distinguishing features include serum triglyceride (TG) levels exceeding 11.3 mmol/L or falling within the range of 5.65–11.3 mmol/L and chylous serum [6]. Although most patients with HTG-AP experience mild disease and can be successfully cured, some may develop local and/or systemic complications, including SIRS and organ failure (OF). OF lasting for more than 48 h is called SAP [12], which is closely related to the prognosis of the disease [13].

Over recent decades, although AP treatment has evolved in a multidisciplinary, individualised, and minimally invasive direction, with improvements in both treatment and care, SAP-related mortality remains as high as 20–40% [1]. Conversely, compared with AP induced by other causes, patients with HTG-AP exhibit a younger age distribution [12]. Furthermore, patients with HTG-AP are more likely to develop hypertriglyceridemia severe acute pancreatitis (HTG-SAP), which has an incidence of 18.2–25.5% [3, 4, 13, 14]. Therefore, early evaluation of the risk of critical illness in HTG-AP is particularly important, especially within 24 h of admission, which is considered a pivotal timeframe to determine the risk of complications or death and implement proactive preventive measures [15].

Currently, recognised AP scoring systems include the Ranson score [16], bedside index for severity in acute pancreatitis (BISAP) [17], acute physiology and chronic health evaluation II (APACHE II) [18], harmless acute pancreatitis score, Japanese severity score (JSS) [19], modified computed tomography (CT) severity index (MCTSI) [20], and the Balthazar rating [21]. These scoring systems have been widely used in clinical practice and exhibit several drawbacks. For example, the APACHE II score has several indicators and is complex to operate, the Ranson score requires more than 48 h to yield results, and the BISAP has poor sensitivity [22]. Furthermore, some studies have shown that the existing AP scoring system has limited value in predicting the severity and prognosis of HTG-AP [23]. Some scholars have attempted to use single biological indicators, including C-reactive protein (CRP), procalcitonin (PCT), blood urea nitrogen (BUN), haematocrit (HCT), and serum macrophage migration inhibition factor to predict the severity of HTG-AP. Although the application is simple, the accuracy is often compromised by the combination of diseases or AP aetiology types, and some indicators are expensive to detect [24]. Additionally, the pathogenesis and pathophysiology of HTG-AP remain unclear, which may be related to the toxic effect of free fatty acids and the influence of lipoglobule acid on pancreatic microcirculation [25]. This differs from AP caused by other aetiologies; therefore, the prognostic biomarkers may also be different. However, there is no clinical multifactor scoring system specific to HTG-AP. For the first time, this study developed a 24 h prognosis model for HTG-AP patients with high morbidity and severity rate, so as to detect the trend of disease intensification early and provide early warning for clinical front-line treatment. At the same time, a variety of statistical methods and R language software were used to explore and screen the predictors of HTG-SAP, and establish a visual model for early prediction of HTG-SAP. Consequently, this study aimed to retrospectively analyse medical records of patients with HTG-AP and screen and explore independent disease predictors along with multiple readily available indicators to develop a prediction model of HTG-SAP, as well as validate the model to improve the prediction of disease severity within 24 h of admission and aid clinical decision-making.

## Methods

### Aim and study design

This study retrospectively analysed the medical records of patients with HTG-AP with the aim to screen for independent risk and protective factors closely related to the severity of HTG-AP and detect markers suggestive of disease progression and prognosis within 24 h of admission. The study aimed to improve the treatment effect of the disease, predict disease severity more accurately, and provide a reference for clinical treatment. Ultimately, this study was conducted to achieve early identification of its tendency to become severe, early intervention, and a reduction in mortality.

### Study participants

Overall, 287 patients with HTG-AP who were hospitalised at the Gastroenterology Department of a Grade A tertiary hospital in Xiamen between January 2019 and December 2021 were selected. Data collected included pre-hospital (emergency, outpatient) and in-patient medical records. The Ethics Committee of Zhongshan Hospital, Xiamen University, approved this study (xmzsyyky Ethics No. 2023 − 139), and the requirement for informed consent was waived.

### Inclusion criteria

Patients were included if they met the following study inclusion criteria: (1) met the AP diagnostic criteria in the Guidelines for Diagnosis and Treatment of Acute Pancreatitis in China (2021) formulated by the Pancreatic Surgery Group of the Chinese Medical Association Surgery Society [5]; (2) had TG ≥ 11.3 mmol/L, or TG ≥ 5.65 and chylous serum; (3) underwent relevant examinations, including CT of the abdomen, pancreas, and chest, completed within 24 h after admission, and no other important observation indicators were missing; and (4) abdominal imaging showed no biliary calculi or obstruction.

### Exclusion criteria

Patients were excluded if they met the following exclusion criteria: (1) received systematic treatment in other hospitals, including but not limited to fluid resuscitation, plasma exchange, and other treatments, prior to admission; (2) acute or chronic diseases of the heart, coronary heart disease, chronic obstructive pulmonary disease, liver cirrhosis, and chronic renal failure; (3) complications with haematological or psychoneurotic diseases; (4) other clear aetiological types of AP, such as biliary, trauma, drugs, and abdominal surgery; (5) presence of clear or suspected infection elsewhere; (6) acute onset of chronic pancreatitis; and (7) incomplete clinical data.

According to the above inclusion and exclusion criteria, 21 patients were excluded from the study due to chronic renal insufficiency (one case of mild disease), appendix abscess (one case of mild disease), anaemia (three cases of mild disease), previous treatment in other hospitals (five cases of mild disease, three cases of moderate-severe disease, and five cases of severe disease), and incomplete clinical data (three cases). Ultimately, 266 patients with HTG-AP were included.

### Severity classification

According to the Atlanta classification criteria [8], patients were classified as mild (MAP; n = 180), moderately severe (MSAP; n = 44), severe (SAP; n = 42), and critical AP (n = 0) with or without persistent OF, pancreatic/systemic infection, and local or systemic complications. Subsequently, the patients were divided into the HTG-SAP (N = 42) and hypertriglyceridemia non-severe acute pancreatitis (HTG-NSAP; N = 224) groups.

### Data collection

The general data of the two groups collected included sex, age, body mass index (BMI), history of alcohol consumption before the onset of disease, history of ordinary alcohol consumption, history of underlying diseases (diabetes mellitus and fatty liver), co-morbidities (diabetic ketoacidosis), number of episodes of pancreatitis, onset of disease to hospitalisation, hospitalisation time, and hospitalisation costs. The data were analysed by considering the highest or lowest values of symptoms, signs, and laboratory tests within 24 h of admission, including the abdominal pain score, presence or absence of psycho-behavioural or mental abnormalities, systolic and diastolic blood pressure (SBP and DBP), pulse rate (P), respiratory rate (R), white blood cell count (WBC), haemoglobin (Hb), HCT, platelet count, SIRS, number of items that meet the SIRS diagnostic criteria, amylase (AMY), lipase (LPS), the maximum value of albumin (ALBMax), the minimum value of albumin (ALBMin), difference between maximum and minimum albumin (dALB), total bilirubin (TBIL), alanine aminotransferase (ALT), aspartate aminotransferase (AST), TG, total cholesterol (TCHOL), blood glucose (GLU), lactate dehydrogenase (LDH), BUN, serum creatinine (Cr), bicarbonate ion (HCO3-), blood calcium (Ca2+), CRP, D-dimer (D-D), PCT, pH, arterial partial pressure of carbon dioxide, arterial partial pressure of oxygen, oxygenation index, blood base residual, plasma lactic acid, and interleukin-6. In this study, the numeric rating scale (NRS) was used. The NRS is a simple scale employed to evaluate pain intensity. Patients were asked to rate their pain degree on a scale ranging from 0 to 10, with 0 indicating no pain and 10 indicating the most severe pain. This scale is routinely used in assessments for patients with pancreatitis after admission. Three days before admission, the doctor-in-charge is responsible for measuring the pain score of patients daily.

The imaging data of all patients within 24 h of admission were reviewed. CT images of the abdomen, pancreas, and chest were re-reviewed, and the conditions of abdominal effusion and pleural effusion were collected. Additionally, severity scores, including the Ranson score (0–11 points), BISAP (0–5 score), and JSS prognostic index (0–9 score) were also collected.

### Statistical analysis

SPSS 26.0 and R 4.1.2 software were used for statistical analyses. Among the collected laboratory test results, the following indicators were deleted due to missing values greater than 10%: BMI, acidity, arterial carbon dioxide partial pressure, arterial oxygen partial pressure, oxygenation index, blood alkali residual, plasma lactate, and interleukin-6.

The one-way analysis of variance (ANOVA) was performed first. Normally distributed measures were expressed as means ± standard deviation, and comparisons between groups were conducted using the independent sample t-test. Non-normally distributed measures were expressed as medians (lower quartile, upper quartile) [M (Q_L_, Q_U_)], and comparisons between groups were performed using the Mann–Whitney rank-sum test. Counts were expressed as the numbers of cases and percentages. The chi-square test was used for comparisons between groups. *P* < 0.05 was considered statistically significant.

Subsequently, the candidate predictors were further selected using the least absolute shrinkage and selection operator (LASSO) regression model. They were included in the binary logistic regression equation for multifactor analysis. The resulting independent predictors for HTG-SAP were used to build the regression model.

R 4.1.2 software was used to plot the column plots of the predicted HTG-SAP models; receiver operator characteristic (ROC) curves were used to determine and compare the area under the curve (AUC), the optimal cut-off value, and the sensitivity corresponding to the optimal cut-off value for the independent predictors and the predictive models. The sensitivity, specificity, positive predictive value (PPV), and negative predictive value (NPV) corresponding to the best cut-off value were calculated. ROC curves of the model and the BISAP, MCTSI, Ranson score, and JSS were established, and the AUC of the model was compared to that of BISAP, MCTSI, Ranson score, and JSS to determine and compare the AUC of each independent predictor and predictive model. To assess the discriminative ability of the model, the Hosmer–Lemeshow test was conducted, and the decision curve analysis (DCA) of the HTG-SAP model was plotted to assess the clinical practicability of the model. Finally, the Bootstrap method was used to repeat the sampling 1000 times for internal validation.

## Results

### Single factor analysis

#### General characteristics of the HTG-NSAP and HTG-SAP groups

There were no significant differences in sex, age, pre-onset and regular drinking history, diabetes history, diabetic ketoacidosis, fatty liver, and the time from onset to hospitalisation between the two groups (all *P* > 0.05). However, the incidence of pancreatitis in the HTG-SAP group was lower than that in the HTG-NSAP group, and the length of hospitalisation and hospitalisation cost in the HTG-SAP group were higher than those in the HTG-NSAP group, with statistical significance (all *P* < 0.05). None of the enrolled patients took lipid-lowering drugs regularly before the onset of pancreatitis. Among the 266 enrolled patients, only 20 patients had taken lipid-lowering drugs in the past; however, none of these patients had been using lipid-lowering drugs regularly, at least within 2–4 weeks before the onset of the disease (Table 1).

**Table 1:** General characteristics of the HTG-NSAP and HTG-SAP groups

Variables	HTG-NSAP group (N = 224)	HTG-SAP group (N = 42)	*χ*^2^ /*Z/T*	*P*-value
Sex, n (%)				
Male	181 (80.80)	34 (80.95)	0.001	0.982
Female	43 (19.20)	8 (19.05)		
Age [M (Q_L_, Q_U_)]	41 (35, 48)	41 (33, 46)	-0.628	0.530
BMI [M (Q_L_, Q_U_)]	26.1 (23.2, 29.1)	26.4 (23.1, 29.7)	-0.452	0.652
Pre-onset drinking history, n (%)	46 (20.54)	12 (28.57)	1.339	0.247
Normal drinking history, n (%)	61 (27.23)	15 (35.72)	1.247	0.264
History of diabetes, n (%)	92 (41.07)	19 (45.24)	0.253	0.615
Combined with diabetic ketoacidosis, n (%)	3 (1.34)	1 (2.38)	0.000	1.000
Combined with fatty liver, n (%)	181 (80.80)	37 (88.10)	1.272	0.259
Number of incidents of pancreatitis [M (Q_L_, Q_U_)]	2 (1, 3)	1 (1, 2)	-2.101	0.036
Time from onset to hospitalisation [M (Q_L_, Q_U_)]	18.0 (10.0, 24.0)	16.0 (10.5, 48.0)	-0.837	0.403
Length of hospitalisation [M (Q_L_, Q_U_)]	7 (5, 9)	17 (12, 27)	-0.849	0.000
Cost of hospitalisation [M (Q_L_, Q_U_)]	10274.98 (7515.88, 14000.38)	63060.145 (25727.60, 96236.86)	-9.162	0.000

HTG-NSAP: Hypertriglyceridemia non-severe acute pancreatitis; HTG-SAP: Hypertriglyceridemia severe acute pancreatitis; BMI: Body Mass Index; M: Median; Q_L_: Lower quartile; Q_U_: Upper quartile

#### Clinical parameters of the HTG-NSAP and HTG-SAP groups

There was no significant difference in SBP, DBP, ALBMax, ALT, and BUN between the two groups (*P* > 0.05). In the HTG-SAP group, abdominal pain score, P, R, WBC, Hb, HCT, number of items meeting the diagnostic criteria of SIRS, AMY, LPS, dALB, TBIL, AST, TG, TCHOL, GLU, LDH, Cr, CRP, D-D, PCT, and severity were obtained. The value or level of score or grading (Balthazar grade, MCTSI, BISAP, Ranson score, JSS CT grade, and JSS prognostic indicators), as well as the incidences of mental, behavioural, or psychiatric abnormalities, SIRS, pleural effusion, and abdominal effusion were higher than those in HTG-NSAP group. PLT, ALBMin, HCO^3−^, and Ca^2+^ were all lower than those in the HTG-NSAP group, and the differences were significant (*P* < 0.05) (Table 2).

**Table 2:** Clinical parameters of the HTG-NSAP and HTG-SAP groups

Variables	HTG-NSAP group (N = 224)	HTG-SAP group (N = 42)	*χ* ^2^ *Z/T*	*P*-value
Abdominal pain score [score, M (Q_L_, Q_U_)]	6 (5, 7)	9 (7, 10)	-6.706	0.000
Mental, behavioural or psychiatric abnormalities, n (%)	0 (0.00)	7 (16.67)	32.114	0.000
P (frequency/minute) (mean ± SD)	93.06 ± 17.56	108.45 ± 19.11	-5.140	0.000
R (frequency/minute)	20 (20, 20)	20 (20, 22)	-4.898	0.000
SBP [mmHg, M (Q_L_, Q_U_)]	133 (121, 146)	137 (118, 151)	-0.514	0.607
DBP (mmHg, Mean ± SD)	87.03 ± 13.48	89.12 ± 14.75	-0.910	0.364
WBC [×10^9^/L, M(Q_L_, Q_U_)]	12.920 (10.493, 15.823)	15.210 (13.533, 18.163)	-3.768	0.000
Hb (g/L, M (Q_L_, Q_U_))	158.000 (148.000, 169.000)	171.000 (148.000, 186.250)	-2.960	0.003
HCT (L/L) (mean ± SD)	44 ± 4.10	47.76 ± 6.76	-3.488	0.001
PLT (×10^9^/L) (mean ± SD)	205.96 ± 60.90	177 ± 64.63	2.801	0.005
SIRS, n (%)	160 (71.43)	42 (100)	15.802	0.000
Number of items meeting the diagnostic criteria of SIRS [number, M (Q_L_, Q_U_)]	2 (1, 3)	3 (3, 3)	-5.572	0.000
AMY [U/L, M (Q_L_, Q_U_)]	238.200 (120.850, 501.200)	680.300 (323.250, 1350.550)	-4.930	0.000
LPS [U/L, M (Q_L_, Q_U_)]	495.250 (229.575, 1147.225)	1659.250 (845.225, 2589.225)	-4.817	0.000
ALBMax [g/L, M (Q_L_, Q_U_)]	44.630 (41.833, 47.108)	43.775 (36.575, 50.115)	-1.046	0.296
ALBMin [g/L, M (Q_L_, Q_U_)]	38.100 (35.600, 40.200)	32.300 (29.725, 36.500)	-6.191	0.000
dALB (g/L, mean ± SD)	6.54 ± 3.97	10.76 ± 5.96	-4.401	0.000
TBIL [µmol/L, M (Q_L_, Q_U_)]	16.300 (12.200, 22.000)	19.200 (14.700, 35.400)	-2.486	0.013
ALT [U/L, M (Q_L_, Q_U_)]	31.600 (21.425, 45.375)	27.400 (17.500, 44.175)	-1.114	0.265
AST [U/L, M (Q_L_, Q_U_)]	28.050 (19.100, 42.150)	53.400 (25.225, 79.050)	-3.901	0.000
TG [mmol/L, M (Q_L_, Q_U_)]	23.500 (13.045, 35.598)	37.755 (22.028, 56.500)	-3.917	0.000
TCHOL [mmol/L, M (Q_L_, Q_U_)]	8.605 (6.398, 11.633)	11.150 (8.448, 15.568)	-3.246	0.001
GLU [mmol/L, M(QL,QU)]	10.450 (7.633, 15.453)	16.055 (11.503, 19.368)	-4.678	0.000
LDH [U/L, M (Q_L_, Q_U_)]	248.350 (203.025, 315.700)	557.300 (402.675, 807.525)	-8.087	0.000
BUN [mmol/L, M (Q_L_, Q_U_)]	4.575 (3.645, 5.720)	4.900 (3.825, 6.793)	-1.450	0.147
Cr [µmol/L, M (Q_L_, Q_U_)]	73.550 (61.325, 86.600)	81.500 (67.400, 107.325)	-2.281	0.023
HCO^3−^ [mmol/L, M (Q_L_, Q_U_)]	23.000 (20.600, 24.500)	17.050 (14.050, 20.625)	-6.877	0.000
Ca^2+^ [mmol/L, M (Q_L_, Q_U_)]	2.200 (2.110, 2.260)	1.805 (1.543, 2.010)	-7.646	0.000
CRP [mg/L, M (Q_L_, Q_U_)]	90.625 (26.343, 162.343)	246.360 (205.800, 312.850)	-7.945	0.000
D-D [µg/L, M (Q_L_, Q_U_)]	0.555 (0.280, 1.310)	2.190 (1.075, 5.018)	-6.729	0.000
PCT [ng/L, M (Q_L_, Q_U_)]	0.110 (0.060, 0.280)	1.215 (0.438, 4.173)	-7.406	0.000
Pleural effusion, n (%)	27 (12.05)	23 (54.76)	42.265	0.000
hydrops abdominis, n (%)	19 (8.48)	33 (78.57)	110.475	0.000
Balthazar classification [score, M (Q_L_, Q_U_)]	2 (2, 3)	4 (4, 5)	-9.072	0.000
MCTSI [score, M (Q_L_, Q_U_)]	2 (2, 2)	4 (2, 6)	-8.898	0.000
BISAP [score, M (Q_L_, Q_U_)]	1 (0, 1)	1 (1, 2)	-7.170	0.000
Ranson score [score, M (Q_L_, Q_U_)]	1 (1, 2)	4 (3, 4)	-8.331	0.000
JSS CT classification [score, M (Q_L_, Q_U_)]	1 (1, 1)	2 (1, 2)	-8.207	0.000
JSS	1 (0, 2)	4 (3, 5)	-9.191	0.000

HTG-NSAP: Hypertriglyceridemia non-severe acute pancreatitis; HTG-SAP: Hypertriglyceridemia severe acute pancreatitis; P: Pulse; R: Respiratory; SBP: Systolic blood pressure; DBP: Diastolic blood pressure; WBC: White blood cell; Hb: Haemoglobin; HCT: Haematocrit; PLT: Platelet; SIRS: Systemic inflammatory response syndrome; AMY: Amylase; LPS: Lipase; ALBMax: The maximum value of albumin; ALBMin: The minimum value of albumin; dALB: Difference between the maximum and minimum values of albumin; TBIL: Total bilirubin; ALT: Alanine aminotransferase; AST: Aspartate aminotransferase; TG: Triglyceride; TCHOL: Total cholesterol; GLU: Glucose; LDH: Lactate dehydrogenase; BUN: Blood urea nitrogen; Cr: Serum creatinine; HCO^3−^: Bicarbonate ion; Ca^2+^: Serum calcium; CRP: C-reactive protein; D-D: D-dimer; PCT: Procalcitonin; MCTSI: Modified CT severity index; BISAP: Bedside index for severity in acute pancreatitis; JSS: Japanese severity scale; M: Median; Q_L_: Lower quartile; Q_U_: Upper quartile; SD: standard deviation

### Independent predictors of HTG-SAP

Indicators with meaningful differences between the two groups in the univariate analysis were selected, which included the number of episodes of pancreatitis, abdominal pain score, mental, behavioural or psychiatric abnormalities, P, R, WBC, Hb, HCT, PLT, number of items meeting the diagnostic criteria for SIRS, AMY, LPS, ALBMin, dALB, TBIL, AST, TG, TCHOL, GLU, LDH, Cr, HCO^3−^, Ca^2+^, CRP, D-D, PCT, presence of SIRS and pleural and abdominal effusion, and the Balthazar classification. The above 30 indicators were included in the LASSO regression analysis to create 1000 models, from which the model with a relatively simple Lambda value of 0.08602453 with a small error and relatively simple composition was selected as a reference. It comprised a total of six indicators, namely, CRP, LDH, Ca^2+^, PCT, the presence or absence of peritoneal effusion, and the Balthazar grading. Subsequently, they were included in the multivariate logistic regression analysis, which revealed that PCT and Balthazar CT grading were equal to *P* > 0.05. Finally, four independent predictors of HTG-SAP, including CRP, LDH, Ca^2+^, and the presence or absence of ascites, were obtained (Table 3). Among them, CRP, LDH, and the presence of peritoneal fluid were independent risk factors, while Ca^2+^ was an independent protective factor.

**Table 3:** Multivariate logistic regression analysis results of independent predictors

Predictive factor	Regression coefficient	Wald	*P-*value	OR	95% CI
CRP	0.008	7.527	0.006	1.008	1.002 ~ 1.013
LDH	0.005	8.359	0.004	1.005	1.002 ~ 1.008
Ca^2+^	-2.804	4.343	0.037	0.061	0.004 ~ 0.846
Ascites	1.701	8.801	0.003	5.481	1.781 ~ 16.866
Constant	0.044	0.000	0.989	1.045	-

HTG-SAP: Hypertriglyceridemia severe acute pancreatitis; CRP: C-reactive protein; LDH: Lactate dehydrogenase; Ca^2+^: Serum calcium

### Establishing a new HTG-SAP prediction model

**Fig. 1:**
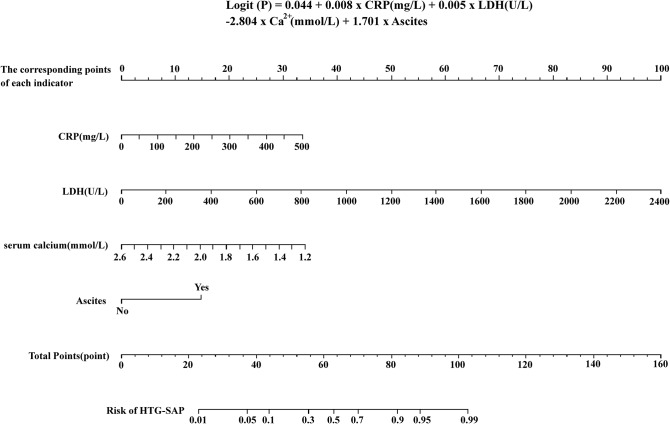
The nomogram for the early prediction model for hypertriglyceridemia severe acute pancreatitis (HTG-SAP). Points are assigned to patients based on the value of C-reactive protein (mg/L), lactate dehydrogenase (U/L), serum calcium (mmol/L), and ascites presence (top two to five lines) by finding the appropriate points on the ‘C-reactive protein’, ‘Lactate dehydrogenase’, ‘Serum calcium’, and ‘Ascites’, and then projecting a vertical line to the ‘Points’ scale at the top line of the nomogram. Subsequently, these points are added together, and the corresponding score on the “Total Points” scale is marked. A vertical line is then projected from the ‘Total Points’ scale to the ‘Risk of HTG-SAP’

According to the results of the multifactor analysis, the logistic regression equation was obtained, as shown in formula (1) (Fig. 1), where 1 indicated the presence of abdominal fluid within 24 h of admission, and 0 was considered otherwise. The HTG-SAP prediction model is presented as a nomogram (Fig. 1).

**Fig. 2:**
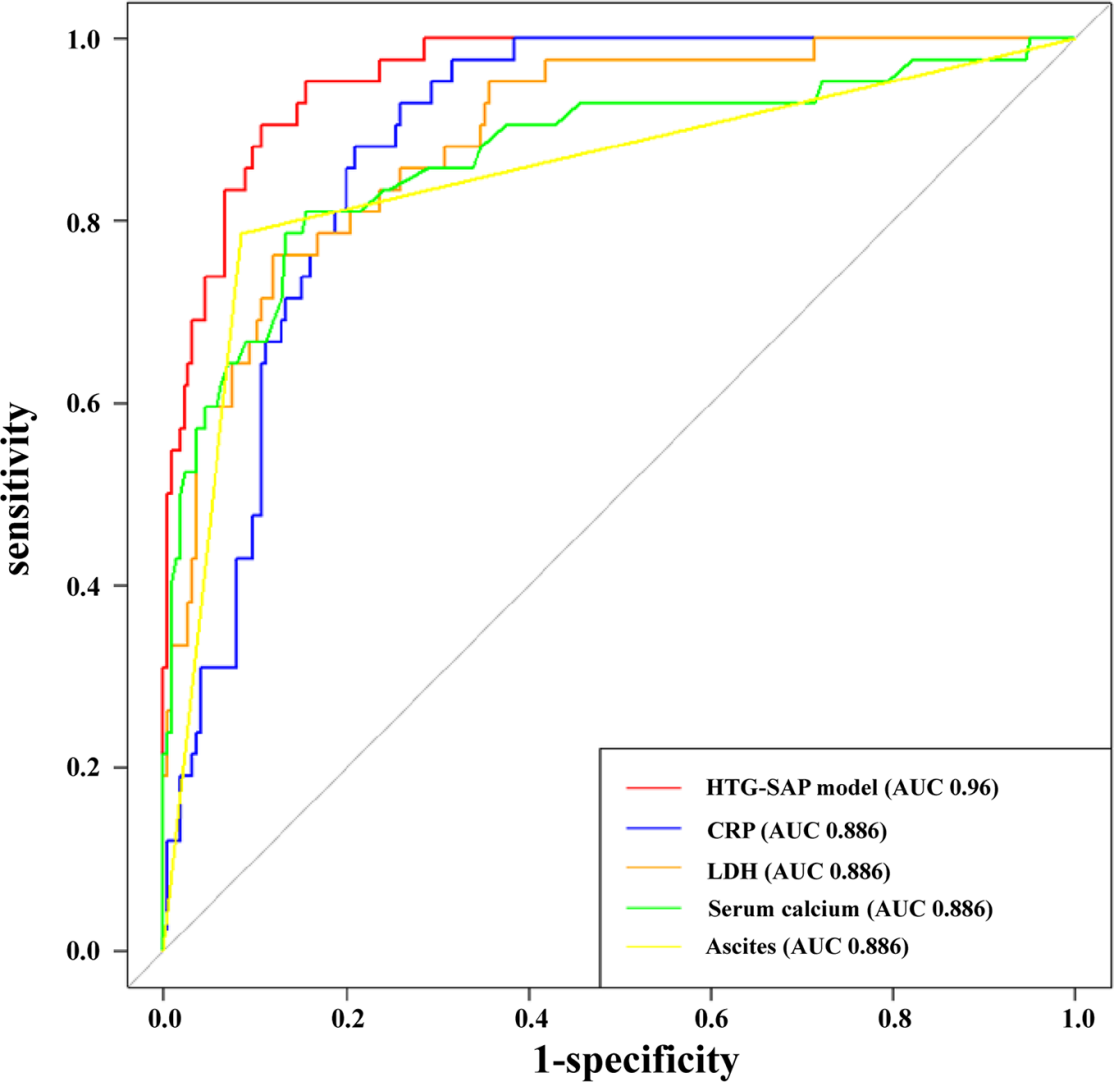
Receiver operator characteristic curve of independent predictors and predictive models for hypertriglyceridemia severe acute pancreatitis (HTG-SAP). The area under the curve (AUC) of the HTG-SAP model, C-reactive protein (CRP), lactate dehydrogenase (LDH), serum calcium, and ascites were 0.960, 0.886, 0.893, 0.872, and 0.850, respectively

The AUCs for CRP, LDH, Ca^2+^, ascites, and the HTG-SAP prediction model were 0.886, 0.893, 0.872, 0.850 and 0.960, respectively. These AUCs were further compared with the HTG-SAP prediction model, and the differences were significant (Z = 3.973, 3.161, 3.043 and 3.996, respectively; *P* < 0.01). The cut-off value, sensitivity, specificity, PPV, and NPV of each independent predictor were calculated using the ROC curves (Table 4; Fig. 2).

**Table 4:** Comparison of independent predictors and predictive models for HTG-SAP

Index	AUC	Cut-off value	Sensitivity	Specificity	PPV	NPV	*P*-value	
CRP	0.886	169.6 mg/L	0.881	0.790	0.440	0.973	< 0.01	
LDH	0.893	407.9 U/L	0.762	0.879	0.542	0.952	< 0.01	
Serum calcium	0.872	2.045 mmol/L	0.810	0.844	0.493	0.959	< 0.01	
Ascites	0.850	0.5	0.786	0.915	0.635	0.958	< 0.01	
HTG-SAP model	0.960	0.152	0.905	0.892	0.613	0.980	-	

AUC: Area under the curve; PPV: Positive predictive value; NPV: Negative predictive value; HTG-SAP: Hypertriglyceridemia severe acute pancreatitis; CRP: C-reactive protein; LDH: lactate dehydrogenase

### Evaluating the HTG-SAP prediction model

#### Assessing the discriminatory power and consistency of models

**Fig. 3:**
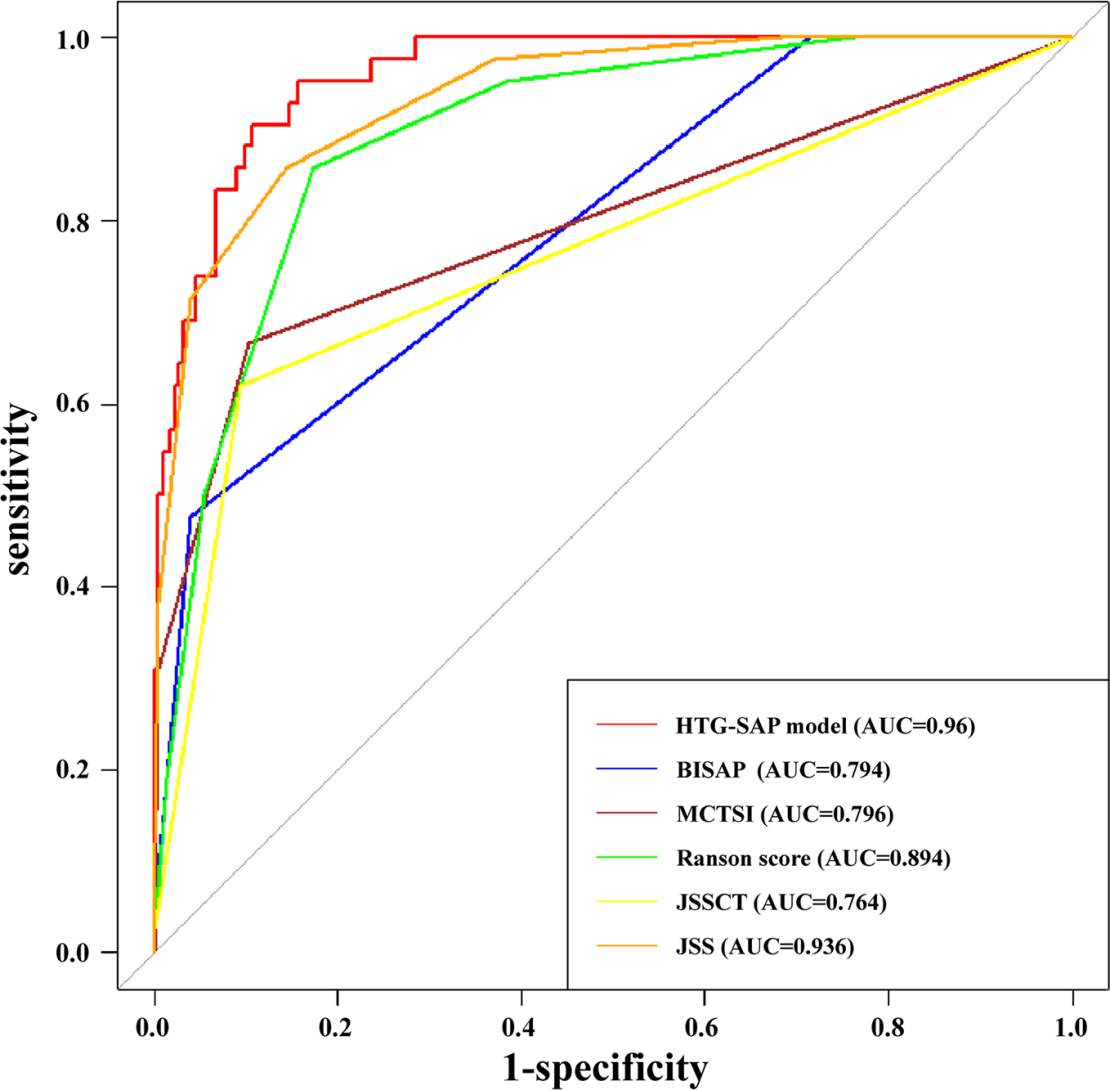
Receiver operator characteristic curve of model for hypertriglyceridemia severe acute pancreatitis (HTG-SAP), bedside index for severity in acute pancreatitis (BISAP), modified CT severity index (MCTSI), Ranson score, and Japanese severity scale (JSS). The area under the curve (AUC) of the HTG-SAP model, BISAP, MCTSI, Ranson score, JSS CT grade, and JSS prognostic factors score were 0.960, 0.794, 0.796, 0.894, 0.764, and 0.936, respectively

The ROC curves of the HTG-SAP prediction model, BISAP, MCTSI, Ranson score, and JSS showed that the ability of the new model to predict the progression of patients to HTG-SAP was better than that of the BISAP, MCTSI, Ranson score, and JSS CT grading. The AUC values of the five models were 0.960, 0.794, 0.796, 0.894, and 0.764, respectively, and the differences were all significant (Z = 5.992, 4.580, 2.842, and 5.509, respectively; *P* < 0.01) (Fig. 3). Although the new model predicted HTG-SAP better than the JSS prognostic indicator, which had an AUC value of 0.936 (95% CI: 0.900–0.972), the difference between the two was not significant (Z = 1.512, *P* = 0.130). Moreover, the Hosmer–Lemeshow test demonstrated that the model achieved a good fit (*P* > 0.05), indicating that the new model’s predicted occurrence probability of HTG-SAP corresponds to the actual probability of HTG-SAP.

#### Assessing the clinical utility of the model

**Fig. 4:**
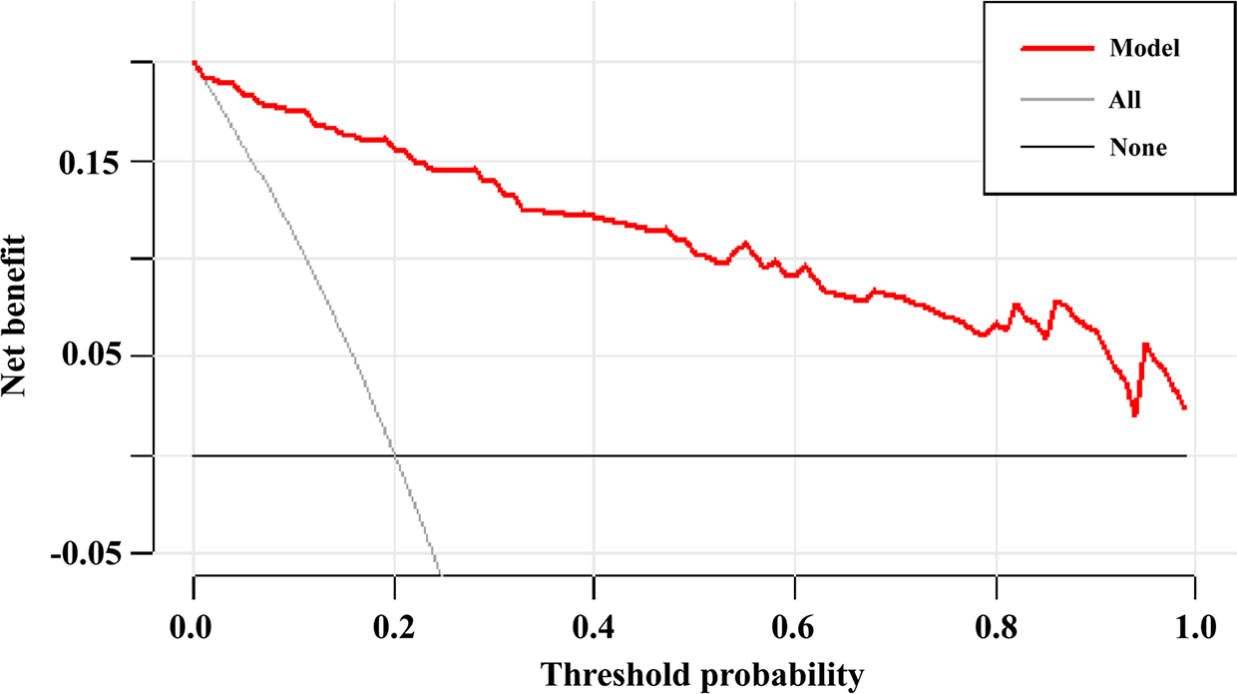
Decision curve analysis for the hypertriglyceridemia severe acute pancreatitis (HTG-SAP) model. The y-axis measures the net benefit, and the x-axis shows the threshold probability. The horizontal black line along the x-axis represents the assumption that no patient will need treatment for HTG-SAP, whereas the solid grey line represents the assumption that all patients will need treatment for HTG-SAP. The red line indicates the HTG-SAP model

Plotting the DCA of the HTG-SAP model revealed that when the threshold probability was greater than 0, the model curve was higher than the two extreme lines, indicating that when the model predicts that patients are at risk of HTG-SAP, timely clinical interventions can prove beneficial and have good clinical value (Fig. 4).

### Internal validation of the models

**Fig. 5:**
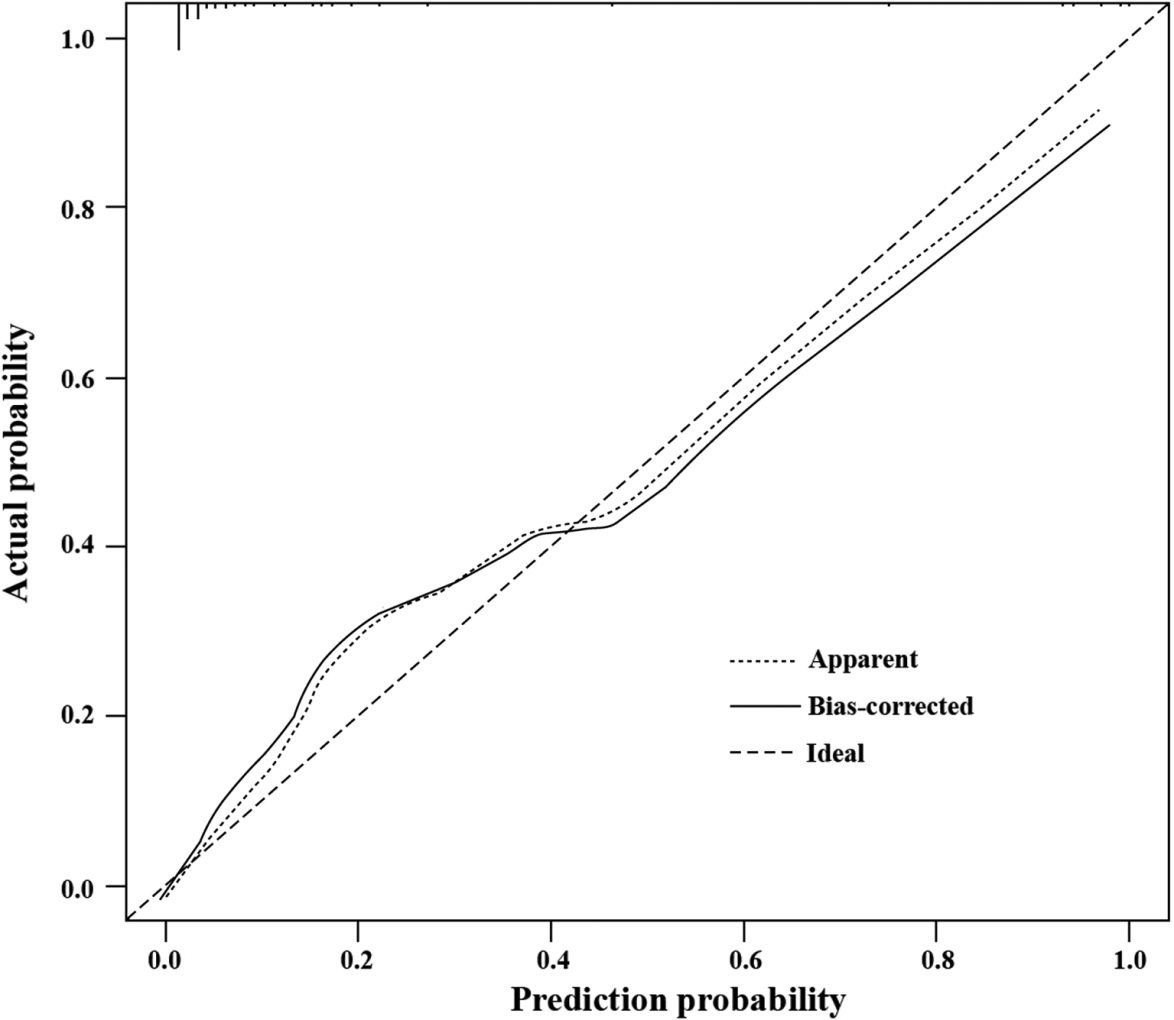
Calibration curve of hypertriglyceridemia severe acute pancreatitis (HTG-SAP) model. The x-axis represents the predicted probability of HTG-SAP calculated according to the model, while the y-axis exhibits the actual probability of HTG-SAP. The apparent calibration curve (dotted line) indicates the model performance in the original data, while the bias-corrected curve (solid line) represents the model performance after correction for optimism using 1000 bootstrap resamples. A perfect prediction would fall on the 45-degree (dashed) reference line

Using the Bootstrap method, the model was repeatedly sampled 1000 times for internal verification. Even after calibration, the high accuracy of the model remained, and the AUC value was 0.955. The calibration curve showed that the original curve was similar to the calibration curve, and both predicted HTG-SAP well (Fig. 5).

## Discussion

AP is prevalent worldwide, and its incidence is rising [26, 27]. The disease course is complex and variable; thus, prediction at early onset is challenging [2]. HTG-AP is the second leading cause of AP. Owing to increasing research on HTG-AP recently, the epidemiology, clinical manifestations, auxiliary examination, and treatment measures of HTG-AP have been identified; however, its pathogenesis remains poorly understood [14, 25]. The clinical significance of early and accurate HTG-SAP identification and providing appropriate and timely treatment lies in hindering the disease course and improving prognosis. Based on common and accessible clinical indicators and imaging studies, 266 cases of HTG-AP from a single clinical centre were retrospectively analysed. The gold-standard grouping was derived from the RAC classification results. Univariate analysis, LASSO regression, and binary logistic regression were used sequentially. Among the 30 candidate predictors, CRP, LDH, Ca^2+^, and ascites presence were identified as independent predictors of HTG-SAP. Four widely recognised AP prognostic scoring systems were compared to the prediction model of HTG-SAP to confirm its consistency and clinical practicability; the results confirmed its accuracy in assessing HTG-SAP occurrence.

In the early stages of HTG-SAP, this study demonstrated that CRP significantly increased. CRP is an acute phase reactant synthesised by the liver, induced by IL-6 and other cytokines [28]. It is a non-specific inflammatory marker and is widely utilised in the diagnosis, prognosis, treatment follow-up, and mortality prediction of various inflammatory or infectious diseases. However, the pathophysiological changes of HTG-AP are closely related to the inflammatory response [29]. Recent domestic and foreign guidelines have highlighted that CRP level ≥ 150 mg/L on the third day of onset can be a prognostic indicator of SAP [15, 30]. Additionally, a CRP increase of > 90 mg/L after admission or > 190 mg/L within 48 h of admission has also been considered a threshold [31]. The results of this study suggest that CRP is an independent predictor of HTG-SAP. The optimal cut-off value from the ROC curve was 169.6 mg/L, the sensitivity was 0.881, and the specificity was 0.790, which is consistent with previous reports.

LDH, a cytoplasmic enzyme that catalyses the conversion of glycolysis-derived pyruvate into lactic acid, is widely expressed in various tissues, including myocardium, skeletal muscle, kidney, pancreas, and tumour tissues, among others and is often used as an indicator of cell death [32]. During the development of HTG-AP, increased LDH may relate to ischemic necrosis of pancreatic acinic cells and AP-related OF, including acute liver and kidney injury. Cui et al. reported that, at an LDH threshold of 647 U/L, the AUC for predicting persistent OF occurrence is 0.876 (95% CI: 0.767–0.985), and the sensitivity and specificity are 76.2% and 98.8%, respectively [33]. Another study involving 153 patients with AP demonstrated that LDH level ≥ 273.04 U/L had good predictive power for SAP and an AUC of 0.919 [34]. Both studies demonstrated that LDH is an independent risk factor for SAP, which is consistent with the results of this study. Uniquely, the present study only discussed and analysed AP with HTG as the aetiological type. Multifactor analysis showed that the regression coefficient of LDH was positive, indicating that LDH is an independent risk factor for HTG-SAP. The optimal threshold for predicting HTG-SAP with LDH alone was 407.9 U/L, the AUC value was 0.893, and the sensitivity was only 76.2%.

Ca^2+^ overload is considered the central link in the pathogenesis of AP [2]. Yu et al. confirmed that the serum Ca^2+^ level of patients with HTG-SAP was lower than that of SAP cases of alternative aetiology [1.69 (95% CI: 1.46–1.91) vs. 2.1 (95% CI: 1.93–2.23), *P* < 0.001] [12]. The current commonly used AP prognostic models, such as the Ranson score and JSS, also include Ca^2+^ in the scoring criteria. Therefore, the inclusion of Ca^2+^ as a predictor in the model is a reasonable choice, especially in the HTG-SAP model.

Ascites are caused by peritoneal infiltration of pancreatic secretions, capillary wall injury, and plasma extravasation [35]. A prospective study showed that patients with AP and ascites exhibited significantly higher rates of OF, severity scores, and mortality and that ascites contributed to the progression of intra-abdominal hypertension to some extent [36]. Zeng et al. reported that patients with ascites were at higher risk for severe disease and worse prognosis, and ascites presence was a risk factor for local and systemic AP complications [37]. Furthermore, by identifying the process by which the MCTSI was established, peritoneal effusion, whether before or after revision, was observed to receive a higher severity score [20]. Previous studies have shown that the early onset of ascites in AP is an important marker of disease severity and is a predictor of local complications [35]. In this study, ascites presence was included as an independent predictor of HTG-SAP, and in addition to being consistent with previous studies, AP blood biological markers and imaging findings were integrated into the same model, increasing the reliability and stability of the model.

This study revealed no statistically significant difference in BUN levels between the two groups, which is inconsistent with previous reports [30, 38]. Another study, which only considered HTG-AP, revealed similar results [39], which may be attributable to the particular pathophysiological changes of HTG-AP or the deviation caused by the small sample size of HTG-SAP cases. This needs to be addressed in studies with a larger sample size.

The HTG-SAP model not only focused on HTG-AP cases and validated its feasibility through diverse statistical methods but also obtained score results within 24 h of admission, which facilitates early detection of the disease severity trend in AP, aiming to provide timely warning and assistance for clinical diagnosis and treatment. The AUC value of the HTG-SAP model constructed in the present study was 0.960, and the 95% CI was 0.936–0.983; this was higher than those of the BISAP, MCTSI, Ranson score, JSS CT grade, and JSS prognostic index (AUCs: 0.794, 0.796, 0.894, 0.764, and 0.936, respectively), indicating that the model had a good ability to distinguish and predict HTG-SAP.

APACHE II [18] is a commonly used and accurate evaluation method. Since not all patients with AP can complete blood gas analysis clinically, approximately 20% of cases in this study lacked blood gas analysis indicators and could not be included in the calculation of the score; thus, the new model was not compared with the APACHE II score. This reflects the disadvantages of complex APACHE II scoring parameters and challenging calculations but also indicates that this study’s HTG-SAP model warrants further verification and/or revision in prospective studies. Wu et al. analysed 1,848 AP cases and found that the sensitivity of BISAP to predict SAP was only 64.9% (95CI: 61.2–68.5%) [40]. The HTG-SAP model established in this study not only has high accuracy but also high sensitivity (90.5%) and specificity (89.2%).

Regarding the Ranson scoring, the drawback is its 48-h completion time and the potential to miss the valuable early treatment window [41]; the model in this present study overcomes this shortcoming. Although the JSS CT grading and MCTSI evaluation require enhanced CT results, existing domestic and international guidelines highlight that if enhanced CT is performed within 72 h after symptom onset, it may underestimate or misclassify the disease severity; therefore, neither is suitable for early AP prediction [2, 5, 15]. The HTG-SAP model only requires the conduction of abdominal CT on the day of the hospital visit to understand abdominal fluid accumulation, which is a necessary examination for patients with AP as the main diagnosis on admission. The current study demonstrated a comparable ability of the new model to predict the risk of HTG-SAP to that of JSS prognostic indicators [AUC: 0.960 (95% CI: 0.936–0.983) vs. 0.936 (95% CI: 0.900–0.972), *P* = 0.130]. Furthermore, the new model requires fewer indicators, and the evaluation process is simpler.

### Study strengths and limitations

The strength of the developed HTG-SAP model is that it comprises three biological indicators for the detection of venous blood samples and requires the completion of abdominal or pancreatic CT scans, which are routine in hospitals and can be performed even in primary care settings. It buys valuable time for the rescue of critically ill patients, which makes the clinical application of this study’s HTG-SAP model broader. This study has some limitations. Firstly, due to the retrospective nature of this study, patient’s subjective symptoms (including abdominal pain score and mental changes) could only be judged by the medical records, and some important indicators (including blood gas analysis) could not be analysed due to lack of medical records for some patients, which may affect the accuracy and completeness of the information; thus, there is information bias. Secondly, this is only a single-centre study, and the disease characteristics of HTG-AP are greatly affected by region, race, and living habits [25]; therefore, it is necessary to expand the research scope further to promote and apply this model. Thirdly, the proportion of severe patients was low (only 42 cases), and although this may relate to the relatively low prevalence rate of HTG-SAP, it is still necessary to expand the sample size to ensure the accuracy of the results. Moreover, the PPV of the new model was 61.3%, which may result in misdiagnosis, excessive medical treatment, and waste of medical resources. Finally, these data lack external validation.

Summarily, it is necessary to further optimise and validate the model in a large sample, multi-centre, prospective cohort. Presently, the prediction and treatment of HTG-SAP pose a significant challenge. With the gradual deepening of AP pathophysiology research and the emergence of new prediction methods, it is believed that the disease trend of HTG-AP will eventually be grasped, thereby empowering clinicians to implement accurate and individualised treatment to reduce disease mortality.

## Conclusions

This study retrospectively analyzed the medical records of patients with HTG-AP, screened out independent risk factors and protective factors closely related to the severity of the disease, and established a prediction model for early severity, so as to provide early warning of markers suggestive of disease progression and prognosis within 24 h of admission, so as to improve the treatment effect of the disease. It can predict the severity of the disease more accurately, provide reference for clinical treatment, and finally achieve the diagnosis and treatment goals of early identification, and intervention, reducing mortality and the tendency of severe disease. CRP, LDH, Ca^2+^, and peritoneal effusion are independent predictors of HTG-SAP. The prediction model created based on these four indicators has high accuracy, sensitivity, consistency, and practicability in predicting HTG-SAP, which will be helpful for clinicians to promptly determine, appropriately diagnose, and treat the disease to improve its prognosis. However, before translating the findings into clinical practice, prospective validation of the predictive value of the HTG-SAP model is required.

### Electronic supplementary material

Below is the link to the electronic supplementary material.

**Supplementary Material 1**: Certificate of Language Editing

**Supplementary Material 2**: Plagiarism Check

## Data Availability

The datasets are available from the corresponding author upon reasonable request.

## List of abbreviations

AP: Acute pancreatitis
HTG-AP: Hypertriglyceridaemia-induced acute pancreatitis
HTG-SAP: hypertriglyceridaemia severe acute pancreatitis
HTG-NSAP: hypertriglyceridaemia non-severe acute pancreatitis
LASSO: least absolute shrinkage with selection operator
P: Pulse
R: Respiratory
SBP: Systolic blood pressure
DBP: Diastolic blood pressure
WBC: White blood cell
Hb: Haemoglobin
HCT: Haematocrit
PLT: Platelet
SIRS: Systemic inflammatory response syndrome
AMY: Amylase
LPS: Lipase
ALBMax: The maximum value of albumin
ALBMin: The minimum value of albumin
dALB: Difference between the maximum and minimum values of albumin
TBIL: Total bilirubin
ALT: Alanine aminotransferase
AST: Aspartate aminotransferase
TG: Triglyceride
TCHOL: Total cholesterol
GLU: Glucose
LDH: Lactate dehydrogenase
BUN: Blood urea nitrogen
Cr: Serum creatinine
HCO^3−^: Bicarbonate ion
Ca^2+^: Serum calcium
CRP: C-reactive protein
D-D: D-dimer
PCT: Procalcitonin
MCTSI: Modified CT severity index
BISAP: Bedside index for severity in acute pancreatitis
JSS: Japanese severity scale
APACHE II: acute physiology and chronic health evaluation II
M: Median
Q_L_: Lower quartile
Q_U_: Upper quartile
SD: standard deviation
CI: Confidence interval
DCA: Decision curve analysis
BMI: Body mass index
ER: Endoplasmic reticulum
FFAs: Free fatty acids
