# Supplementary material for: Establishment and validation of early prediction model for hypertriglyceridemic severe acute pancreatitis
Source: Lipids Health Dis. 2023 Dec 8;22:218. doi: 10.1186/s12944-023-01984-z (PMC10709974; doi:10.1186/s12944-023-01984-z)
Supplement: Supplementary file 1 — Supplementary Material 1: Certificate of Language Editing [file 12944_2023_1984_MOESM1_ESM.pdf]

Editing Certificate

This document certifies that the manuscript listed below has been edited to ensure language and grammar accuracy and is error free in these aspects. The logical presentation of ideas and the structure of the paper were also checked during the editing process. The edit was performed by professional editors at Editage, a brand of Cactus Communications. The author's core research ideas were not altered in any way during the editing process. The quality of the edit has been guaranteed, with the assumption that our suggested changes have been accepted and the text has not been further altered without the knowledge of our editors.

MANUSCRIPT TITLE

Establishment and Validation of Early Prediction Model for Hypertriglyceridemic Severe Acute Pancreatitis

AUTHORS

Yi Shuanglian,Zeng Huiling,Lin Xunting,Deng Yifang,Lin Yufen,Xie Shanshan, Si Lijuan, LiuYunpeng

ISSUED ON

November 28, 2023

JOB CODE

YQOCW_2_12

THE EDITAGE Promise QUALITY GUARANTEE

Prabh Grewal
Senior Vice President - Editage

editage | helping you get published

Since 2002, Editage has helped over 430,000 authors publish around 1.2 million research papers in scholarly journals across over 1000 disciplines through editorial, translation, transcription, and publication support services. Editage is a brand of Cactus Communications (cactusglobal.com), a science communication and technology company.

CACTUS

GLOBAL :
+1(833) 979-0061 | request@editage.com

CHINA :
400-120-3020或 021-6020-9400 | fabiao@editage.cn
